# Sex Hormone–Binding Globulin Levels Are Inversely Associated With Nonalcoholic Fatty Liver Disease in HIV-Infected and -Uninfected Men

**DOI:** 10.1093/ofid/ofz468

**Published:** 2019-11-06

**Authors:** Jennifer C Price, Ruibin Wang, Eric C Seaberg, Todd T Brown, Matthew J Budoff, Lawrence A Kingsley, Frank J Palella, Mallory D Witt, Wendy S Post, Jordan E Lake, Chloe L Thio

**Affiliations:** 1 Division of Gastroenterology and Hepatology, Department of Medicine, University of California San Francisco, San Francisco, California, USA; 2 Department of Epidemiology, Johns Hopkins Bloomberg School of Public Health, Baltimore, Maryland, USA; 3 Division of Endocrinology, Diabetes, & Metabolism, Department of Medicine, Johns Hopkins University School of Medicine, Baltimore, Maryland, USA; 4 Division of Cardiology, Department of Medicine, Los Angeles Biomedical Research Institute at Harbor-UCLA Medical Center, Torrance, California, USA; 5 Departments of Infectious Diseases and Microbiology and Epidemiology, University of Pittsburgh Graduate School of Public Health, Pittsburgh, Pennsylvania, USA; 6 Division of Infectious Diseases, Department of Medicine, Northwestern University Feinberg School of Medicine, Chicago, Illinois, USA; 7 Division of HIV Medicine, Department of Medicine, Los Angeles Biomedical Research Institute at Harbor-UCLA Medical Center, Torrance, California, USA; 8 Division of Cardiology, Department of Medicine, Johns Hopkins University School of Medicine, Baltimore, Maryland, USA; 9 Division of Infectious Diseases, Department of Medicine, University of Texas Health Science Center at Houston, Houston, Texas, USA; 10 Division of Infectious Diseases, Department of Medicine, Johns Hopkins University School of Medicine, Baltimore, Maryland, USA

**Keywords:** fatty liver, HIV, NAFLD, SHBG, testosterone

## Abstract

**Background:**

Nonalcoholic fatty liver disease (NAFLD) is a leading cause of liver disease worldwide. Elevated sex hormone–binding globulin (SHBG) levels have been observed in the setting of HIV and may protect against some metabolic disorders. We aimed to investigate whether higher SHBG levels may protect against NAFLD in men with/without HIV.

**Methods:**

NAFLD was assessed using noncontrast computed tomography in 530 men in the Multicenter AIDS Cohort Study (MACS) who drank <3 alcoholic drinks/d and were uninfected with chronic hepatitis C or B (340HIV+, 190HIV-). Morning serum samples were tested for SHBG, total testosterone (TT), and adiponectin. Multivariable logistic regression was used to assess associations between HIV, SHBG, TT, adiponectin, and NAFLD.

**Results:**

Median SHBG was highest among HIV+/NAFLD- men and lowest among HIV-/NAFLD+ men. Adjusted for demographics, HIV, visceral adiposity, HOMA-IR, TT, and *PNPLA3* genotype, higher SHBG was associated with lower odds of NAFLD (odds ratio [OR], 0.52 per doubling; 95% confidence interval [CI], 0.34–0.80). In separate multivariable models without SHBG, HIV (OR, 0.46; 95% CI, 0.26–0.79) and higher adiponectin (OR, 0.66 per doubling; 95% CI, 0.49–0.89) were associated with lower NAFLD odds, whereas TT was not significantly associated (OR, 0.74 per doubling; 95% CI, 0.53–1.04). Adjusting for SHBG attenuated the associations of HIV (OR, 0.61; 95% CI, 0.34–1.08) and adiponectin (OR, 0.74; 95% CI, 0.54–1.02) with NAFLD.

**Conclusions:**

SHBG levels were higher among HIV+ men, were independently associated with lower NAFLD, and could partially explain the associations of HIV and higher adiponectin with lower NAFLD in our cohort. These findings suggest that SHBG may protect against NAFLD, supporting further prospective and mechanistic studies.

Nonalcoholic fatty liver disease (NAFLD) is a leading cause of liver disease worldwide, with a global prevalence of 25% [1]. Associations of obesity, dyslipidemia, diabetes, and metabolic syndrome with NAFLD are well established [2]. Metabolic alterations are increasingly common among aging HIV-infected persons, and NAFLD is highly prevalent in this group, raising concerns that persons living with HIV infection are at increased risk of NAFLD [3, 4]. However, we previously found that HIV infection was associated with a lower odds of NAFLD among men who participated in the Multicenter AIDS Cohort Study (MACS) [5]. Similarly, in the Women’s Interagency HIV Study, HIV-infected women without viral hepatitis had significantly less liver fat than HIV-uninfected women [6]. The association of less liver fat with HIV infection in these studies was surprising and was not altered after adjusting for multiple potential confounders. Recently, other cohort studies have similarly demonstrated lower rates of fatty liver in people living with HIV compared with those without HIV, for reasons that remain unclear [7, 8].

Sex hormone–binding globulin (SHBG) is a liver-derived protein that may have an important role in energy metabolism. Lower SHBG levels are associated with a higher prevalence of diabetes and metabolic syndrome [9, 10]. Although the mechanisms driving this relationship remain unclear, existing evidence suggests a causal role of SHBG in the development of diabetes. Prospective studies show a protective effect of higher SHBG against incident diabetes, and genetic studies demonstrate that carriers of single nucleotide polymorphisms in *SHBG* that raise SHBG levels have a lower risk of type 2 diabetes, whereas carriers of the SHBG-lowering allele have an increased risk [9, 11, 12]. Moreover, the relationship of SHBG with diabetes is bi-directional, as insulin inhibits hepatic SHBG production [13].

Studies performed to date in HIV-uninfected populations have similarly demonstrated lower circulating SHBG levels in patients with NAFLD, although the directionality of this association is unclear because SHBG is produced in the liver [14]. Low SHBG levels could be a consequence of NAFLD, as fat deposition in the liver may lead to downregulation of SHBG production [15]. However, preclinical models suggest that low SHBG could increase NAFLD risk, as overexpression of SHBG in mice causes decreased lipogenesis and is protective against NAFLD development [16]. Notably, HIV-infected men in the MACS have higher SHBG levels than HIV-uninfected men, despite having more insulin resistance and diabetes [17]. Other studies have similarly observed elevated SHBG levels in association with HIV infection, but the reasons for this are not understood [18–20]. We hypothesized that higher SHBG levels may be protective against NAFLD and may explain the apparent protective association between HIV infection and NAFLD. We tested this hypothesis in the subset of MACS men previously evaluated for NAFLD [5].

## METHODS

### Study Design and Participants

We conducted a cross-sectional analysis within the MACS, an ongoing prospective cohort study of men who have sex with men. MACS participants were recruited from 4 sites in the United States (Baltimore, MD, USA/Washington DC, USA, Chicago, IL, USA, Pittsburgh, PA, USA, and Los Angeles, CA, USA). Details of study recruitment and participant characteristics have been described elsewhere [21, 22]. Participants are followed semi-annually for interviews, physical examination, and laboratory testing. This analysis includes men who were enrolled in the MACS cardiovascular disease substudy and who underwent computed tomography (CT) imaging from January, 2010, to August, 2013 [23]. Exclusion criteria for the cardiovascular disease substudy were age <40 or >70 years, weight >300 pounds, history of cardiac surgery, or history of coronary angioplasty or stent placement. For the current analysis, we additionally excluded men who consumed an average of ≥3 alcoholic drinks per day, were chronically infected with hepatitis C virus (HCV) or hepatitis B virus, had incomplete visualization of the liver and spleen on noncontrast cardiac CT, did not have serum available for sex hormone testing, or were missing other key covariates (Supplementary Figure 1). Among the 603 men who met criteria for inclusion, 530 men had available morning serum samples for sex hormone measurement between 1 year before and 15 days after their CT scan date and were included in the final analysis.

Candidate covariates, including fasting serum insulin, glucose, triglycerides, alanine aminotransferase (ALT), aspartate aminotransferase (AST), CD4^+^ T-cell count, and plasma HIV-1 RNA levels, were obtained from the MACS visit most proximal to the CT scan, as previously described [24]. Age, race, and medications were obtained through self-report. Body mass index (BMI) was calculated as body weight (kg)/height (m)^2^. Diabetes was defined as either fasting glucose ≥126 mg/dL or use of a diabetes medication. The homeostatic model assessment of insulin resistance (HOMA-IR) was natural log–transformed. Hypertension was defined as any of the following: systolic blood pressure (BP) >140 mmHg, diastolic BP >90 mmHg, or use of an antihypertensive medication. *PNPLA3* (rs738409) genotype was determined in our prior study [5]. The study was approved by the local ethical committee at each MACS site, and all participants signed informed consent.

### Fatty Liver and Adipose Tissue Measurements

Fatty liver was defined as a liver/spleen Hounsfield unit ratio <1.0 on noncontrast CT scan, as previously described [5]. CT scans were reviewed by a single reader who was blinded to participant demographic and clinical data. A single CT slice in the space between the fourth and fifth lumbar vertebrae was used to measure visceral adipose tissue (VAT) and subcutaneous adipose tissue (SAT) areas [25].

### Sex Hormone and Adiponectin Measurements

All hormone assays were performed on morning serum samples stored at –80°C from the MACS visit closest to CT scanning. Testing was performed on 334 samples in 2010 using the DELFIA immunofluorometric assay (Turku, Finland) and on the remaining 196 samples in 2017 using the Beckman Coulter Access Chemluminescent Immunoassay (Fullerton, CA, USA). Total testosterone (TT) levels were measured using liquid chromatography tandem mass spectrometry on all 530 samples. Adiponectin levels were measured from serum samples stored at –80°C and collected at the time of CT scanning using enzyme-linked immunosorbent assay (R&D Systems, Minneapolis, MN, USA).

### Statistical Analysis

We compared participant characterisitics by HIV serostatus using the chi-square test for categorical variables and the Wilcoxon rank-sum test for continuous variables. The Kruskal-Wallis test was used to compare SHBG values across HIV and NAFLD categories. To determine if SHBG or TT was associated with NAFLD, multivariable logistic regression models with sex hormone levels as the primary predictors and NAFLD as the outcome were constructed in a sequential fashion: adjusted for age, race, MACS site, test batch (to adjust for the 2 different SHBG assays), HIV serostatus, *PNPLA3* genotype (base model), base model + VAT area, and base model + VAT area + HOMA-IR. The model covariates were selected based on our prior analyses of factors associated with NAFLD in the cohort [5, 24]. SHBG and TT levels were log_2_-transformed and were first evaluated individually in the regression models and then included jointly in the models. The final model included both SHBG and TT levels after formally assessing for multicollinearity using the variance inflation factor, which was within the acceptable range [26]. We repeated the analysis stratified by HIV serostatus to determine whether the observed relationship of SHBG levels with NAFLD differed by HIV serostatus. To determine whether adjustment for SHBG levels attenuates the magnitude of the association of HIV infection with NAFLD, we used a base model that adjusted for HIV serostatus, age, race, MACS site, test batch, and *PNPLA3* genotype and evaluated HIV serostatus as the primary predictor with and without SHBG in the model. Finally, we repeated this process, adding adiponectin levels (which we previously found to be inversely associated with NAFLD regardless of HIV serostatus [24]) or TT to the base model with/without SHBG. Statistical analyses were performed using Stata/SE, version 13.1 (StataCorp, College Station, TX, USA), and R, version 3.3.2 (The R Foundation for Statistical Computing, Vienna, Austria).

## RESULTS

### Study Population

Compared with the 190 HIV-uninfected men, the 340 HIV-infected men were slightly younger and had a lower median SAT area and median BMI but a similar median VAT area (Table 1). The HIV-infected men also had lower high-density lipoprotein cholesterol levels, higher triglyceride levels, and higher ALT and AST levels compared with the HIV-uninfected men. Median SHBG and median TT levels were significantly higher among HIV-infected compared with HIV-uninfected men. Median adiponectin levels were lower in the HIV-infected compared with HIV-uninfected men, but this was not statistically significant. The HIV-infected men had lower NAFLD prevalence compared with HIV-uninfected men (15% vs 21%; *P* = .086).

**Table 1.** Characteristics of the Study Population

Characteristic	HIV-Infected (n = 340), Median (IQR) or No. (%)	HIV-Uninfected (n = 190), Median (IQR) or No. (%)
Age, y	**52 (48–57)**	**54 (50–62)**
Race		
White non-Hispanic	195 (57)	127 (67)
Black non-Hispanic	104 (31)	43 (23)
Other	41 (12)	20 (11)
BMI, kg/m^2^	**26 (23–29)**	**27 (24–30)**
Abdominal VAT, mm^2^	150 (88–218)	148 (92–212)
Abdominal SAT, mm^2^	**179 (114–265)**	**226 (163–304)**
Diabetes	37 (11)	20 (11)
HOMA-IR	3.2 (2.2–4.9)	3.0 (2.2–4.2)
On lipid-lowering agent	129 (39)	60 (32)
HDL cholesterol, mg/dL	**46 (37–54)**	**48 (41–58)**
LDL cholesterol, mg/dL	107 (85–136)	110 (90–134)
Triglycerides, mg/dL	**131 (93–205)**	**107 (77–154)**
Hypertension	160 (49)	78 (43)
ALT, U/L	**25 (19–35)**	**22 (17–28)**
AST, U/L	**23 (20–31)**	**22 (18–25)**
APRI >1.5	3 (1)	0 (0)
FIB-4 >3.25	**9 (3)**	**0 (0)**
*PNPLA3* non-CC genotype	138 (41)	89 (47)
NAFLD on CT scan	50 (15)	39 (21)
Adiponectin	5984 (3684–9725)	6539 (4444–9491)
SHBG	**53 (36–71)**	**39 (28–52)**
Total testosterone	**588 (432–767)**	**474 (355–666)**
Recent viral load <50 copies/mL	283 (83)	
Recent CD4+ T-cell count, cells/µL	597 (438–779)	
Cumulative HAART, y	9 (6–12)	

Bold formatting signifies statistical significance at *P* < .05.

Abbreviations: ALT, alanine aminotransferase; APRI, aspartate aminotransferase to platelet ratio index; AST, aspartate aminotransferase; BMI, body mass index; FIB-4, fibrosis-4 index; HAART, highly active antiretroviral therapy; HDL, high-density lipoprotein; HOMA-IR, homeostatic model assessment of insulin resistance; IQR, interquartile range; LDL, low-density lipoprotein; NAFLD, nonalcoholic fatty liver disease; SAT, subcutaneous adipose tissue; SHBG, sex hormone–binding globulin; VAT, visceral adipose tissue.

### SHBG But Not Total Testosterone Is Associated With Lower NAFLD

**Figure 1.**
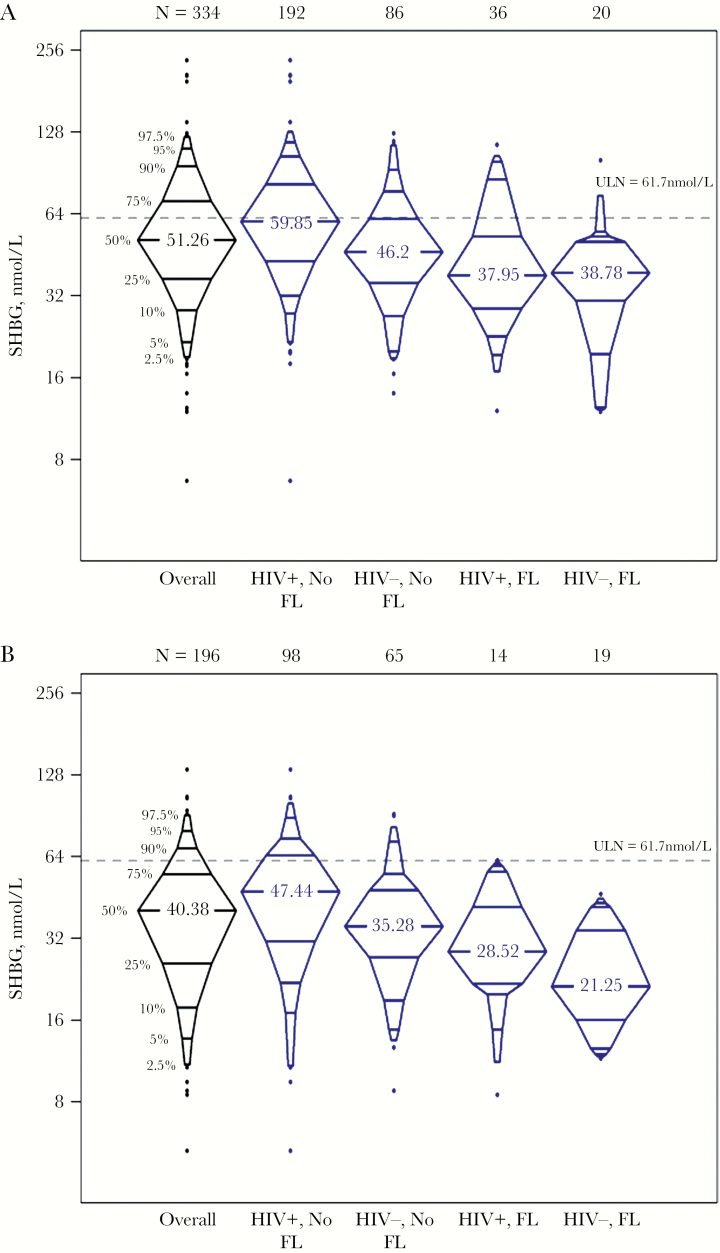
SHBG levels by HIV serostatus and NAFLD status. A, SHBG measured using the DELFIA assay. B, SHBG measured using the Beckman Coulter assay. Abbreviations: FL, fatty liver; SHBG, sex hormone–binding globulin.

SHBG levels were highest among HIV-infected men without NAFLD, followed by HIV-uninfected men without NAFLD, and were lowest among men with NAFLD regardless of HIV serostatus (Figure 1). Median SHBG values among the 334 men with measurement using the DELFIA assay were HIV+/NAFLD- 60.0 nmol/L, HIV-/NAFLD- 46.2 nmol/L, HIV+/NAFLD+ 38.0 nmol/L, and HIV-/NAFLD+ 38.8 nmol/L (*P* < .001). Median SHBG values among the 196 men with measurement using the Beckman Coulter assay were HIV+/NAFLD- 47.4 nmol/L, HIV-/NAFLD- 35.3 nmol/L, HIV+/NAFLD+ 28.5 nmol/L, and HIV-/NAFLD+ 21.3 nmol/L (*P* < .001). TT and SHBG levels were positively correlated with each other (Spearman *rho* correlation coefficient = .52; *P* < .001). Hence, the pattern of TT level distribution after stratifying by HIV and NAFLD status was similar to that observed with SHBG (data not shown).

In multivariable analysis, higher SHBG levels were associated with lower odds of NAFLD (OR, 0.38 per SHBG doubling; 95% confidence interval [CI], 0.26–0.56) (Table 2). This association was attenuated but remained significant after further adjusting for VAT and HOMA-IR (OR, 0.52; 95% CI, 0.34–0.80). The addition of BMI to the model did not change the association. To determine if the association was independent of TT, the model including VAT and HOMA-IR was run excluding TT, and the association of SHBG with lower NAFLD remained (OR, 0.50; 95% CI, 0.34–0.75). Notably, TT levels were not associated with NAFLD in any model, including a model with VAT and HOMA-IR (OR, 0.93; 95% CI, 0.63–1.36) (Table 2), and even in a model excluding SHBG (OR, 0.74; 95% CI, 0.52–1.04) (Table 3). These analyses were repeated stratified by HIV serostatus. The relationship of SHBG with NAFLD was similar between the groups, and likewise, we did not find an association of TT levels with NAFLD (Supplementary Table 1).

**Table 2.** Association Between SHBG Levels and NAFLD

	Model 1^a^		Model 1^a^ + Visceral Adiposity		Model 1^a^ + Visceral Adiposity + HOMA-IR	
	Odds Ratio (95% CI)	*P*	Odds Ratio (95% CI)	*P*	Odds Ratio (95% CI)	*P*
Log2 (SHBG)	**0.38 (0.26–0.56)**	**<.001**	**0.50 (0.33–0.77)**	**.001**	**0.52 (0.34–0.80)**	**.003**
Log2 (TT)	0.75 (0.53–1.07)	.12	0.87 (0.60–1.25)	.44	0.93 (0.63–1.36)	.70
HIV infection	0.93 (0.55–1.58)	.80	0.65 (0.37–1.15)	.14	0.61 (0.34–1.09)	.10
VAT (per 10 mm^2^)			**1.07 (1.04–1.11)**	**<.001**	**1.05 (1.02–1.09)**	**.002**
Ln (HOMA-IR)					**1.91 (1.17–3.11)**	**.01**
Age (per year)	1.00 (0.96–1.04)	.82	0.97 (0.93–1.01)	.14	0.97 (0.93–1.01)	.13
Black race	**0.26 (0.12–0.54)**	**<.001**	**0.42 (0.19–0.92)**	**.03**	**0.34 (0.15–0.77)**	**.01**
*PNPLA3* non-CC	**1.81 (1.10–2.98)**	**.02**	**2.03 (1.20–3.41)**	**.01**	**2.17 (1.28–3.68)**	**.004**

Bold formatting signifies statistical significance at *P* < .05.

Abbreviations: CI, confidence interval; HOMA-IR, homeostatic model assessment of insulin resistance; NAFLD, nonalcoholic fatty liver disease; SHBG, sex hormone–binding globulin; TT, total testosterone; VAT, visceral adipose tissue.

^a^Also adjusted for MACS sites and test batch.

**Table 3.** Association of HIV Infection, Total Testosterone Levels, and Adiponectin Levels With NAFLD, With and Without Adjustment for SHBG Levels

	Odds Ratio (95% CI)	
	Without SHBG^a^	With SHBG
Log2 (SHBG)	–	**0.52 (0.34–0.80)**
Log2 (TT)	0.74 (0.53–1.04)	0.93 (0.63–1.36)
Log2 (SHBG)	–	**0.50 (0.34–0.75)**
HIV infection	**0.46 (0.26–0.79)**	0.61 (0.34–1.08)
Log2 (SHBG)	–	**0.56 (0.37–0.85)**
Log2 (Adiponectin)	**0.66 (0.49–0.89)**	0.74 (0.54–1.02)

Bold formatting signifies statistical significance at *P* < .05.

Abbreviations: CI, confidence interval; HOMA-IR, homeostatic model assessment of insulin resistance; NAFLD, nonalcoholic fatty liver disease; SHBG, sex hormone–binding globulin; TT, total testosterone; VAT, visceral adipose tissue.

^a^Base model includes HIV serostatus, age, race, MACS site, testing batch, *PNPLA3* genotype, VAT, and HOMA-IR. TT and adiponectin were each evaluated separately in the multivariable models.

### Adjusting for SHBG Attenuates the Association of HIV and NAFLD

After adjusting for age, race, MACS site, testing batch, *PNPLA3* genotype, VAT area, and HOMA-IR, HIV serostatus was associated with lower prevalence of NAFLD (OR, 0.46; 95% CI, 0.26–0.79), consistent with our prior analysis (Table 3) [5]. Interestingly, after adjustment for SHBG levels, HIV infection was no longer significantly associated with lower NAFLD odds (OR, 0.61; 95% CI, 0.34–1.08), whereas associations of VAT, HOMA-IR, black race, and *PNPLA3* non-CC genotype with NAFLD were unchanged (Table 2).

### Adjusting for SHBG Adjustment Attenuates the Association of Adiponectin and NAFLD

We previously found an inverse relationship between levels of adiponectin, an adipokine involved in modulating glucose and lipid metabolism, and NAFLD in our cohort, regardless of HIV serostatus [24]. In the present analysis, we again found that higher levels of adiponectin were associated with lower NAFLD odds in a multivariable analysis (OR, 0.66 per doubling; 95% CI, 0.49–0.89) (Table 3). This association was no longer statistically significant after further adjusting for SHBG levels (OR, 0.74; 95% CI, 0.54–1.02). However, higher levels of SHBG remained independently associated with lower NAFLD odds even after adding adiponectin to the model (OR, 0.50 and 0.56 with and without adiponectin, respectively) (Table 3).

## DISCUSSION

In this study of 530 well-characterized men with or at risk for HIV infection, higher SHBG levels were associated with lower odds of NAFLD after adjusting for known metabolic and genetic risk factors, as well as for levels of TT and adiponectin. This finding was consistent regardless of HIV serostatus. Further, SHBG levels may explain, at least in part, the observed association between HIV and less NAFLD in our cohort, as this association was attenuated after accounting for SHBG levels.

SHBG is a protein produced in the liver that binds testosterone and other sex hormones and regulates their bioavailabilty [27]. SHBG may also have functions beyond transporting sex hormones, including maintaining metabolic homeostasis [28]. To our knowledge, this study is the first to evaluate the association of SHBG levels with NAFLD in the setting of HIV infection. Our finding of an inverse relationship between SHBG levels and NAFLD has been reported previously in studies of HIV-uninfected persons. In a meta-analysis of 5 studies conducted among HIV-uninfected men, high SHBG levels were associated with decreased NAFLD odds (pooled OR, 0.35; 95% CI, 0.25–0.45) [14]. SHBG levels have also been inversely associated with metabolic perturbations that are linked to NAFLD, including insulin resistance, diabetes, and central adiposity [17, 29, 30]. Notably, in our current report, higher SHBG levels remained strongly associated with lower odds of NAFLD, even after adjusting for HOMA-IR and VAT area. Hence, our observed association of SHBG levels with NAFLD is unlikely to be a result of residual confounding exerted by metabolic factors.

Although associations of higher SHBG levels with decreased NAFLD could be due to reduced hepatic production of SHBG in the presence of liver fat [15, 31, 32], there are several lines of evidence supporting the assertion that SHBG actually protects against NAFLD. First, adiponectin, which is one of the most important adipokines as it regulates body weight, insulin sensitivity, and inflammatory status [33, 34], was no longer associated with NAFLD after accounting for SHBG levels. In contrast, the SHBG association with NAFLD remained, independent of adiponectin levels; this suggests that SHBG mediates the protective effect of adiponectin on NAFLD. Second, recent evidence suggests that SHBG levels protect against NAFLD development by modulating lipogenesis. In genetic and diet-induced NAFLD mouse models, overexpression of SHBG leads to significant reductions in liver fat by downregulating key lipogenic enzymes [16]. Similarly, treatment with exogenous SHBG in vitro inhibits lipogenesis by decreasing PPAR-γ levels, whereas underexpression of SHBG results in increased hepatic lipogenesis [16]. Third, single nucleotide polymorphisms in *SHBG* that downregulate SHBG levels are associated with development of type 2 diabetes, suggesting that SHBG is causal [11]. Fourth, a study examining gene expression pathways from 72 liver samples from persons with NAFLD identified SHBG as 1 of 5 leading candidates for affecting NAFLD development [35].

It is possible that the observed relationship of SHBG and NAFLD is due to testosterone rather than SHBG. However, TT levels were only weakly associated with NAFLD in our study, and adjusting for SHBG levels further weakened the association. Although a meta-analysis of 10 studies found an inverse relationship between TT levels and NAFLD among HIV-uninfected men [14], the analysis did not evaluate the combined associations of SHBG and TT levels with NAFLD. One study reported an inverse association of TT and NAFLD in unadjusted analysis that was diminished after adjusting for other NAFLD risk factors and SHBG [36]. Another study directly measured free testosterone (FT) and SHBG and found a strong inverse association between SHBG and NAFLD but failed to find an association between FT and NAFLD [37]. Finally, the protective effect of SHBG against NAFLD development persists in castrated mouse models, supporting our findings that TT is not driving the association of SHBG with NAFLD [16]. We were unable to assess the independent relationship between FT levels and NAFLD in this study because FT was not directly measured but rather calculated based on TT and SHBG levels. In addition, ~9% of the men in our cohort were on testosterone supplementation. Thus, it is unclear whether our findings are generalizable to people with hypogonadism.

Despite the adverse metabolic effects of HIV infection and some antiretroviral medications, our prior analysis of MACS men unexpectedly found a protective association between HIV infection and prevalent NAFLD [5], a finding that is reproduced in the subset of men included in this analysis. We now further demonstrate that SHBG may partially mediate this protective association between HIV infection and NAFLD, as HIV infection was no longer significantly associated with protection from NAFLD after accounting for SHBG levels. Further work is needed to ascertain the mechanisms by which HIV infection may increase SHBG levels. Interestingly, patients with active HCV also have elevated SHBG levels, which decline after achievement of sustained virologic response (SVR) with HCV treatment [38]. In a Canadian prospective cohort study, hepatic steatosis progressed faster in HIV-monoinfected patients compared with HIV/HCV-coinfected patients, and in another cohort of adults with and without HIV and HCV, non–genotype 3 HCV was independently associated with less liver fat despite adjustment for multiple factors, including liver fibrosis [39, 40]. It is intriguing to consider whether SHBG may mediate the association of active HCV with less hepatic steatosis and whether the post-SVR decline in SHBG may affect a patient’s risk of developing NAFLD after SVR.

The major strengths of our study were the large sample size with comprehensive assessments of multiple NAFLD risk factors, such as visceral adiposity, insulin resistance, and *PNPLA3*, and the inclusion of an HIV-uninfected comparator group. However, a major limitation is that we were unable to assess the directionality of the relationship between SHBG and NAFLD because of the cross-sectional design. Another limitation was the reliance on noncontrast CT to diagnose fatty liver; noncontrast CT will detect moderate or greater amounts of liver fat but is less sensitive to detect mild fatty liver. We also do not have nonfasting measures of insulin resistance in our cohort and therefore could not assess whether insulin levels throughout the day affect the association of SHBG and NAFLD. In addition, the MACS is comprised only of men, and therefore our findings may not be generalizable to women living with or at risk for HIV infection. This is especially important because the association of sex hormones and NAFLD appears to differ by sex [14]. Finally, we did not have liver biopsies available and were therefore unable to evaluate whether SHBG levels correlate with histologic severity of liver disease in our cohort. This is important because HIV-infected individuals with NAFLD may have an elevated risk of nonalcoholic steatohepatitis and fibrosis [4, 41].

In conclusion, we found that among men living with or at risk for HIV infection, SHBG levels were inversely associated with NAFLD regardless of HIV serostatus. SHBG levels were higher among HIV-infected men regardless of the presence of NAFLD, and adjustment for SHBG levels attenuated the association of HIV infection with lower odds of NAFLD. The underlying mechanisms driving elevations in SHBG levels among HIV-infected persons remain unclear and warrant further investigation. Our findings also support prospective evaluation of the relationship of SHBG and NAFLD and translational research focused on potential mechanisms by which SHBG may influence NAFLD development and progression. Finally, further work is needed to determine the relationship of SHBG, HCV, and NAFLD.

## Supplementary Data

Supplementary materials are available at *Open Forum Infectious Diseases* online. Consisting of data provided by the authors to benefit the reader, the posted materials are not copyedited and are the sole responsibility of the authors, so questions or comments should be addressed to the corresponding author.

ofz468_suppl_Supplemental_Figure_1

ofz468_suppl_Supplemental_Table_1
