# Supplementary figures and images for: Sex Hormone–Binding Globulin Levels Are Inversely Associated With Nonalcoholic Fatty Liver Disease in HIV-Infected and -Uninfected Men
Source: Open Forum Infect Dis. 2019 Nov 6;6(12):ofz468. doi: 10.1093/ofid/ofz468 (PMC7047947; doi:10.1093/ofid/ofz468)

**Supplemental Figure**. Flow chart of study population.


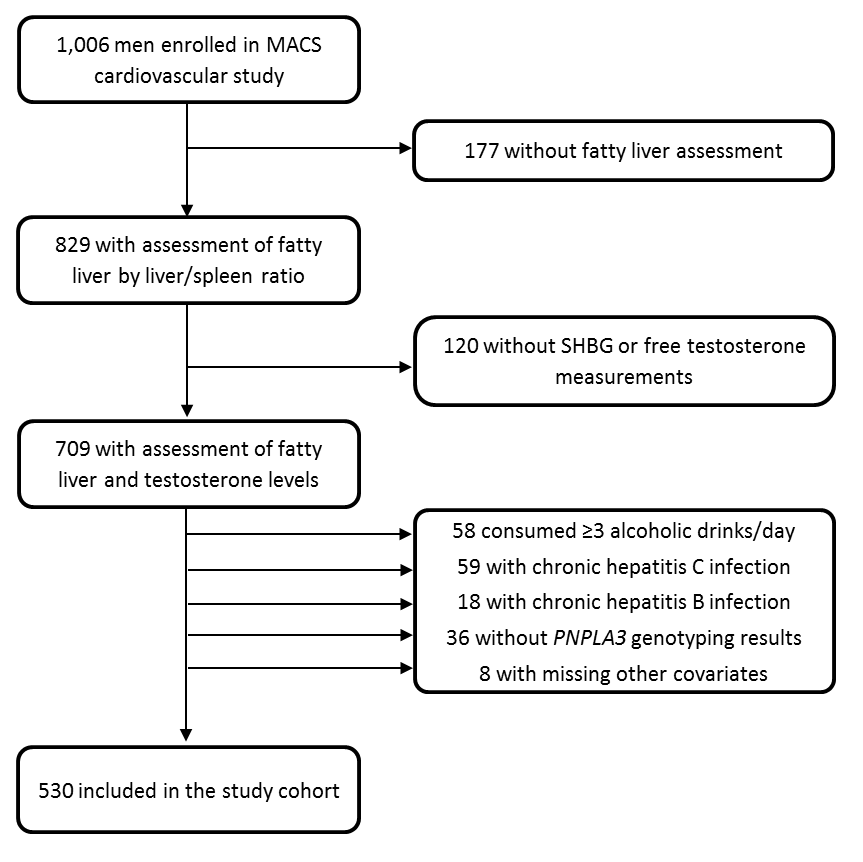

Supplement: ofz468_suppl_Supplemental_Figure_1 [file ofz468_suppl_supplemental_figure_1.docx]
