# Supplementary material for: Sex Hormone–Binding Globulin Levels Are Inversely Associated With Nonalcoholic Fatty Liver Disease in HIV-Infected and -Uninfected Men
Source: Open Forum Infect Dis. 2019 Nov 6;6(12):ofz468. doi: 10.1093/ofid/ofz468 (PMC7047947; doi:10.1093/ofid/ofz468)
Supplement: ofz468_suppl_Supplemental_Table_1 [file ofz468_suppl_supplemental_table_1.docx]

**Supplemental Table.** Association between SHBG levels and NAFLD by HIV serostatus

|  | **HIV+ (N=340)** | | **HIV- (N=190)** | |
| --- | --- | --- | --- | --- |
|  | Odds ratio  (95% CI) | p-value | Odds ratio  (95% CI) | p-value |
| Log2(SHBG) | 0.61 (0.36-1.04) | 0.07 | **0.39 (0.18-0.83)** | **0.01** |
| Log2(TT) | 0.84 (0.51-1.40) | 0.51 | 1.14 (0.61-2.13) | 0.69 |
| VAT (per 10mm^2^) | **1.05 (1.01-1.10)** | **0.02** | **1.07 (1.01-1.13)** | **0.03** |
| Ln(HOMA-IR) | **1.81 (1.01-3.25)** | **0.048** | 1.93 (0.77-4.82) | 0.16 |
| Age (per year) | **0.93 (0.87-0.99)** | **0.03** | 1.01 (0.94-1.07) | 0.84 |
| Black race | **0.36 (0.13-0.98)** | **0.046** | 0.28 (0.06-1.25) | 0.10 |
| *PNPLA3* non-CC | **2.60 (1.30-5.22)** | **0.007** | 1.65 (0.69-3.93) | 0.26 |

Bold signifies statistical significance at p-value<0.05.

P-value for interaction between HIV serostatus and SHBG is 0.49.

*All models were also adjusted for MACS sites and test batch

Abbreviations: CI, confidence interval; HIV, human immunodeficiency virus; HOMA-IR, homeostatic model assessment of insulin resistance; NAFLD, nonalcoholic fatty liver disease; SHBG, sex hormone-binding globulin; TT, total testosterone; VAT, visceral adipose tissue.
